# THE AGE-FRIENDLY UNIVERSITY (AFU) INITIATIVE AND THE CALL FOR MORE AGE-FRIENDLY INTERGENERATIONAL CLASSROOMS

**DOI:** 10.1093/geroni/igac059.446

**Published:** 2022-12-20

**Authors:** Joann Montepare

**Affiliations:** Lasell University, Newton, Massachusetts, United States

## Abstract

Changing age demographics are reshaping societies and challenging our living, learning, and work communities to consider how they can respond to more age-diverse, older populations. The pioneering Age-Friendly University (AFU) initiative, endorsed by GSA’s Academy for Gerontology in Higher Education (AGHE), calls for institutions of higher education to respond to these shifting demographics through more age-friendly programs, practices, and partnerships. Moreover, the 10 AFU Principles recommend a set of specific educational options including the promotion of intergenerational learning to facilitate the reciprocal sharing of expertise between learners of all ages. This presentation provides an overview of the AFU initiative with a focus on strategies for intergenerational teaching and learning. It sets the stage for the other presentations by describing core questions and considerations educators should take into account when mounting intergenerational practices in and beyond the classroom.

